# Nicotine Activates YAP1 through nAChRs Mediated Signaling in Esophageal Squamous Cell Cancer (ESCC)

**DOI:** 10.1371/journal.pone.0090836

**Published:** 2014-03-12

**Authors:** Yue Zhao, Wei Zhou, Liyan Xue, Weimin Zhang, Qimin Zhan

**Affiliations:** 1 State Key Laboratory of Molecular Oncology, Cancer Institute and Hospital, Chinese Academy of Medical Sciences and Peking Union Medical College, Beijing, China; 2 Department of Pathology, Cancer Institute and Hospital, Chinese Academy of Medical Sciences and Peking Union Medical College, Beijing, China; The University of Hong Kong, China

## Abstract

Cigarette smoking is an established risk factor for esophageal cancers. Yes-associated protein 1 (YAP1), the key transcription factor of the mammalian Hippo pathway, has been reported to be an oncogenic factor for many cancers. In this study, we find nicotine administration can induce nuclear translocation and activation of YAP1 in ESCC. Consistently, we observed nuclear translocation and activation of YAP1 by knockdown of CHRNA3, which is a negative regulator of nicotine signaling in bronchial and esophageal cancer cells. Nicotine administration or CHRNA3 depletion substantially increased proliferation and migration in esophageal cancer cells. Interestingly, we find that YAP1 physically interacts with nAChRs, and nAChRs-signaling dissociates YAP1 from its negative regulatory complex composed with α-catenin, β-catenin and 14-3-3 in the cytoplasm, leading to upregulation and nuclear translocation of YAP1. This process likely requires PKC activation, as PKC specific inhibitor Enzastaurin can block nicotine induced YAP1 activation. In addition, we find nicotine signaling also inhibits the interaction of YAP1 with P63, which contributes to the inhibitory effect of nicotine on apoptosis. Using immunohistochemistry analysis we observed upregulation of YAP1 in a significant portion of esophageal cancer samples. Consistently, we have found a significant association between YAP1 upregulation and cigarette smoking in the clinical esophageal cancer samples. Together, these findings suggest that the nicotine activated nAChRs signaling pathway which further activates YAP1 plays an important role in the development of esophageal cancer, and this mechanism may be of a general significance for the carcinogenesis of smoking related cancers.

## Introduction

Cigarette smoking is associated with an increased risk of both squamous-cell carcinoma and adenocarcinoma of the esophagus [1]–[3]. Recent findings suggest nicotine and its derivatives such as NNN (N-nitrosonornicotine) and NNK((4-methylnitrosamino)-1-(3-pyridyl)-1-butanone) can direct activate nicotinic acetylcholine receptors (nAChRs) to stimulate the growth and angiogenesis and suppress the drug induced apoptosis of the cancer cells [4]. Nicotinic acetylcholine receptors (nAChRs) are a family of ligand gate ion channels that function as the major regulators of nicotinic and acetylcholinergic signaling in the cells. α-nAChRs and β-nAChRs are the most common nACHRs. Imbalanced expressions of different subtypes of nAChRs in the cells contribute to the pathogenesis of diseases such as cancer [4]. nAChRs are also known to regulate cellular adhesion and migration through their interactions with rapsyn and herparansulphate proteogly can such as agrin [5]–[7].

Overexpression and increased nuclear localization of hippo pathway transcription factor YAP1have been observed in multiple types of human cancers, including liver cancers, colon cancers, ovarian cancers, lung cancers, and prostate cancers [8]. And amplifications of YAP1 gene locus are observed in intracranial ependymomas, oral squamous cell carcinomas, and medulloblastomas [8]. YAP1 was reported to be the cancer driving gene in the human hepatocellular carcinoma (HCC) and breast cancer 11q22 amplicons [9], [10]. Moreover, YAP1 was determined to be an independent prognostic marker for overall survival of hepatocellular carcinoma and esophageal squamous cell carcinomas [11], [12].

Dasgupta et al. reported nicotine can induce up regulation of XIAP and Survivin (BIRC5) in Non-Small Cell Lung Cancer to inhibit apoptosis induced by chemotherapeutic drugs [13]. And nicotine is known to induce the expression of CTGF (connective tissue growth factor) in gingival fibroblasts and in periodontal ligament cells which contributes to the pathogenesis of periodontal fibrosis [14]. Notably, Survivin and CTGF are two of the conserved downstream genes regulated by transcription factor YAP1 of the Hippo pathway [8], [15]. Recently, Yu et al. reported regulation of the Hippo-YAP pathway by G-protein-coupled receptor signaling [15]. And β2-nAChR were reported to be physically associated with G protein, αG protein-regulated inducer of neurite out growth 1, and G protein-activated K(+) channel 1, indicating a possible link between nAChRs signaling and cellular G protein pathways [16]. Moreover, YAP1 was reported to be upregulated in esophageal cancers and is determined as an oncogene in esophageal cancer [11], [17]. Thus we attempted to explore the possible connection between nicotine exposure and YAP1 activation in esophageal cancer in this study.

## Methods

### Tissue Specimens

Our ESCC tissue specimens from 83 patients with pathological T3 stage esophageal squamous cell carcinoma were collected. Patients were consecutively recruited at the Chinese Academy of Medical Sciences Cancer Hospital (Beijing, China). At recruitment, informed consent was obtained from each subject. The consents were in written form, each patient was informed to sign the consent for using their tissue samples acquired from surgery for science research before the samples were taken. We preserved the consent table in our medical data base, and the ethics committee/IRB of Cancer Institute of Chinese Academy of Medical Sciences approved the consent procedure and the study.

### Cell Culture and Transfection and drug administration

Human ESCC cell lines were cultured in RPMI 1640 medium supplemented with 10% fetal bovine serum at 37°C under 5% CO2 and saturated moisture. Esophageal cancer cell lines KYSE510, KYSE30 were provided generously by Dr. Yutaka Shimada (Kyoto University). For the transient transfections of plasmid and siRNA, cells were grown on 60-mm plates in 50–90% confluence and transfected with 200 p mol of siRNA using Lipofectamine 2000 (Invitrogen). Three Stealth siRNAs targeted to CHRNA3 were designed and ordered from Invitrogen,TureClone plasmid of tGFP-CHRNA3 was purchased from OriGene. Cells were transfected with tGFP-CHRNA3 plasmid or CHRNA3 siRNA using Lipofectamine 2000 (Invitrogen) according to the manufacturer's instruction. Fresh medium was added 6 hours after transfection. For nicotine administration, cells were incubated in medium containing 80 nM of nicotine for 48 hours or longer. For Enzastaurin administration, Enzastaruin was added to the medium at the concentration of 500 µM for 48 hours.

### Cell Growth Curve

Growth curve measured by xCELLigence RTCA MP E-plate 96 well, 3×10^3^ cells were added to each well according to the protocol of xCELLigence System, cell growth rate was monitored for 81 h. For cell growth curves read by MTT assay, 100 µl of cell culture containing 3×10^3^ cells were added to 30 wells of a 96-well plate. 20 µl of methanethiosulfonate reagent (Promega) was added to 6 wells each time at 24 h interval for 5 days, followed with 1 h of incubation at 37°C and 5% CO^2^, the absorbance were read at 490 nm with a microplate reader.

### Transwell Migration and Invasion Assays

Migration and invasion assays were carried out with Corning's 80 µm 24-well transwell plate coated with 30% Matrigel (300 µl/well, Falcon). In total, 1×10^5^ cells in 100 µl of serum-free medium were added to the upper chamber of the device, and the lower chamber was filled with 600 µl of culture medium with 20% fetal bovine serum. After 6 hours of incubation at 37°C, the non-migrating cells were removed from the upper surface of the membrane with a cotton swab. The filters were then fixed in methanol for 10 min, stained with Giemsa solution for 1 hour, and counted. Five random microscopic fields (×100) were counted per well, and the mean was determined.

### Antibodies and Special Reagents

Rabbit YAP1 antibody, CHRNA3 antibody were purchased from Proteintech Gourp (Chicago, IL 60612, USA), mouse YAP (63.7), GAPDH antibody were purchased from Santa Cruz biotechnology (Dallas, Texas 75220U.S.A.), Phospho-YAP (Ser127) antibody and β-catenin antibody, β-actin antibody were purchased from Cell Signaling Technology (Danvers, MA 01923), CHRNB4 antibody, CHRNA5 antibody and 14-3-3 antibody were purchased from Abgent. tGFP antibody, P63 antibody, tGFP labeled CHRNA3 Tureclonevector were purchased from OriGene (Beijing, CHINA 101111), α-catenin antibody was purchased from Lifetechnologies (Carlsbad, CA 92008). Recombinant GST labeled YAP1 protein was purchased from Abnova (Taipei City Taiwan114). PKC inhibitor Enzastaurin was purchased from Selleck Chemicals (Munich, Germany 81829). Nicotine was purchased from Sigma-Aldrich (St. Louis, MO 63103).

### Immunofluorescence assay

KYSE510 cells were seeded on glass coverslips in a 6-well plate for 24–48 hours, cells were fixed with methanol for 10 min at room temperature and washed with PBS. After incubation with associated antibody for 1 hour at room temperature, plates were washed and incu1'bated with FITC-conjugated goat anti-rabbit IgG. After being washed with PBS, cells were stained with DAPI and examined with a laser-scanning confocal microscope (Leica Microsystems Heiderg GmbH, Am Friedensplatz 3, Germany).

### Quantitative Real Time PCR

Total RNA from KYSE510 cells was extracted with TRIzol (Life technologies (Carlsbad, CA 92008)). First-strand cDNA was synthesized by using the Superscript II-reverse transcriptase kit (Invitrogen) according to the manufacturer's instructions. Real-time PCR (qPCR) was conducted using SYBR Premix Ex Taq (Takara) on an ABI 7300 Real-Time PCR System (Applied Biosystems). All samples were normalized to GAPDH.

The gene-specific PCR primer pairs used in this study:

YAP1 (GGCGCTCTTCAACGCCGTCATGAAC/CCTGTCGGGAGTGGGATTT);

CTGF (CCAATGACAACGCCTCCTG/TGGTGCAGCCAGAAAGCTC);

Cyr61 (AGCCTCGCATCCTATACAACC/TTCTTTCACAAGGCGGCACTC);

EDN1 (TGTGTCTACTTCTGCCACCT/CCCTGAGTTCTTTTCCTGCTT);

PPP1ReB (GGACACGTTCTCCTTCGAC/AGATTTTAACTCAGCCCGGAT);

SURVIVIN (CCTGGCTCCTCTACTGTT/CTCTATTCTGTCTCCTCATCC);

GAPDH (GCTGAGAACGGGAAGCTTGT/GCCAGGGGTGCTAAGCAGTT )

### Western Blotting, Immunoprecipitation, and GST Pull-down Assays

Western blot analysis was performed as follows. Cells were collected and centrifuged for harvesting. Cells were lysedon ice for 40 min in RIPA buffer (10 mMTris, pH 7.4, 150 mMNaCl, 1% Triton X-100, 0.1% sodium deoxycholate, 0.1% SDS, and 5 mM EDTA) containing Complete Protease Inhibitor Mixture(Sigma). Lysates were clarified by centrifugation at 12,000relative centrifugal force for 20 min at 4°C. For Western blotting,40 µg of total protein was suspended in sample buffer. For immunoprecipitation, lysates were incubated with primaryantibody followed by incubation with protein A-agarose beads (Invitrogen). The immune complexes were washed and suspendedin sample buffer. In GST pull-down assay, glutathione beads(Sigma) were incubated with Escherichia coli-expressed GST-YAP1or GST alone for 4 hours. Glutathionebeads were then washed and incubated for 4 hours with lysates of KYSE510 cells. After washing, the protein complexes were suspended in sample buffer. All protein was loaded into each wellof a 15% SDS-PAGE. Gels were transferred onto PVDF membranes (Bio-Rad), blocked with 5% milk/PBS, and incubated overnight at 4°C with primary antibodies. Following washing and incubation with secondary antibodies in 5% milk, the membrane was washed, and the positive signals were developed with chemiluminescence reagent (Amersham). The membrane was exposed to medical x-ray film (Fuji Ltd., Tokyo, Japan).

### Flow cytometric analysis

The vital, apoptotic and damaged cells were separated by flow cytometry. The quantitative determination of the percentage of cells undergoing apoptosis was performed using an annexin V-FITC apoptosis detection kit (Cliniscience S.A.) according to the manufacturer's instructions. In brief, 48 hours after treatment with nicotine, 2×10^5^ cells were labeled fluorescently for detection of apoptotic and necrotic cells by adding 195 µl of annexin V binding buffer and 5 µl of annexin V-FITC to each sample. Samples were mixed gently and incubated at room temperature in the dark for 3 min, 10 µl of propidium iodide (PI; Sigma) was added to each sample and incubated at room temperature for 10 min. Before cytometric analysis, the cell suspension was supplemented with 300 µl of annexin V-binding buffer. A minimum of 10,000 cells within the gated region were acquired and analyzed with Cell Quest software.

### Immunohistochemical staining and assessment

All the tissues were fixed in 4% neutralised formaldehyde, embedded in paraffin. Blocks of paraffin-embedded donor tissue were sampled using a Manual Tissue Arrayer 1 instrument (Beecher Instruments, Silver Spring, MA, USA). Two cores were cut from each donor block for the TMA blocks. Sections (5 µm) of the tissue array block were cut and placed on polylysine-coated glass slides and processed for IHC. From the samples available, seven tissue array blocks were prepared, each containing 30 cases with tumor, normal and lymph node tissues if available. The tissue microarray slides were deparaffinized in xylene and gradient ethanol. Antigen retrieval was performed by placing the slides in a high-pressure cooker in a 0.01 mM citrate buffer, pH 6.0, for 2.5 min at 100°C; they were then cooled for 20 min. Endogenous peroxidase activity was blocked by incubating the section in 3% H_2_O_2_ for 10 min, followed by rinsing in PBS solution three times. The sections were incubated with rabbit anti-YAP1 antibody (Proteintech) at a dilution of 1∶50 at 4°C overnight, The slides were then incubated for 1 h in secondary antibody. An EnVision kit (Dako, Carpinteria, CA, USA) was used to visualize antibody binding, and slides were subsequently counterstained with hematoxylin. A PBS-only staining sample was used as a background control. The tissue array slides were scanned and analyzed with AperioScanScope CS. Based on the immunostaining intensity, esophageal tissues were divided into four categories as YAP1 negative (−), weak positivity of YAP1 (+), median positivity of YAP1(+ +), strong positivity of YAP1 (+ + +). All experiments were performed and repeated at least three times. Data were analyzed with SPSS 11.5software. Correlations between the subgroups of staining and cigarette smoking were calculated using the Pearson χ2 test.

## Results

### Nicotine promotes the growth and migration of esophageal cancer

**Figure 1:**
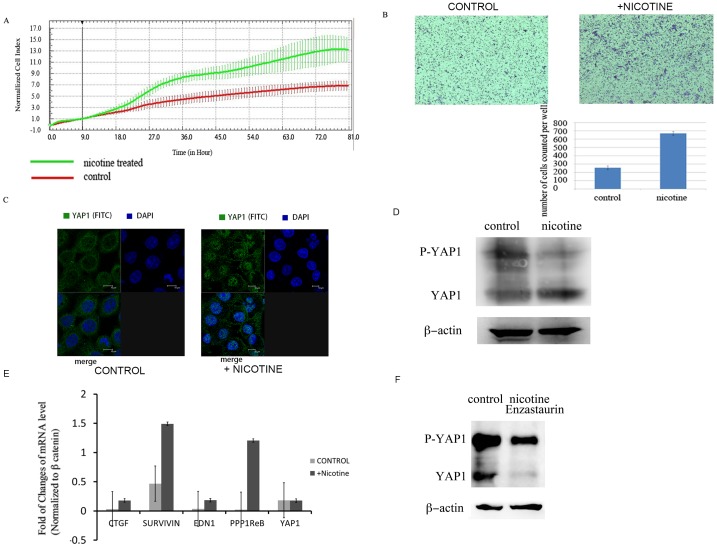
A. Nicotine administration stimulates the growth of esophageal cancer KYSE510 cell measured by E-Plateofx CELLigence RTCA MP system. B. Nicotine administration increased the invasion and migration of esophageal cancer KYSE30 cells in transwell assays. C. Subcellular localization of YAP1 examined with confocal fluorescence microscope. Translocation of YAP1 (green) from the cytoplasm to the nucleus was observed after nicotine administration in KYSE510 cells for 48 h. D. KYSE510 cells were treated with nicotine for 48 hs, decreased phosphorylation of YAP1 and increased dephosphorylated YAP1 was observed by Western blot analysis. E. Real-time PCR verification of induction of mRNAs of genes transcriptionally activated by YAP1 upon nicotine administration. F. PKC inhibitor Enzastaurin blocked nicotine induced upregualtion of YAP1 protein level, and resulted in reduction of YAP protein level, particularly the dephosphorylated form of YAP1 by Western blot.

Nicotine is known to be an important risk factor for esophageal cancer. The previous reports demonstrate that nicotine promotes the cell growth and migration in different types of human cancers [4], [18]. Thus we first tested the growth stimulatory effects of nicotine in esophageal cancer KYSE510 cell line with xCELLigence RTCA MP E-plate 96 well and observed that nicotine administration substantially enhanced the growth rate of KYSE510 cells (Figure 1 A). Additionally, we conducted transwell assay to study the effects of nicotine on the migration of KYSE510 cell and also observed a significant increase of the migration of the KYSE510 cell treated with nicotine (Figure 1 B).

### Nicotine induces YAP1 nuclear translocation and activation

Up regulation and increased nuclear localization of Hippo pathway transcription factor YAP1 was reported to be the independent marker for worse survival of esophageal cancer [11]. To evaluate if nicotine exposure would resulted in the activation of YAP1. We investigated the subcellular localization of YAP1 in esophageal cancer KYSE510 cell using confocal immunofluorescence microscope following exposure to Nicotine. After cells were cultured in the media containing nicotine at the concentration of 80 nM for 48 hours, we observed an increased nuclear translocation of YAP1, as manifested by YAP1 accumulation in the nucleus after cells treated by Nicotine (Figure 1 C). Since nuclear translocation and activation of YAP1 is regulated by the phosphorylation of YAP1 on S127 site [8], we then measured the changes of phosphorylation level of YAP1 before and after nicotine treatment. As shown in Figure 1 D, decreased phosphorylation of YAP1 and increased protein level of dephosphorylated YAP1 were observed after nicotine administration. We further examined mRNA expression of CTGF, a YAP1 targeted downstream gene, and found that mRNA levels of CTGF was elevated by nicotine administration. However we did not observe significant upregulation of YAP1 mRNA after nicotine administration (Figure 1 E). These results suggest that following nicotine administration, YAP1 undergoes nuclear translocation and in turn transcriptionally activates its downstream genes including CTGF.

PKC has been reported to be required for nAChR activation by forming an auto-positive feedback loop for the activation of nicotinic acetylcholine receptors [19], [20]. It has also been identified as a YAP1 kinase [21]. Thus we treated the cells with PKC specific inhibitor Enzastaurin to see if YAP1 activation can be blocked by PKC inhibition. We observed that Enzastaurin treatment substantially blocked YAP1 activation induced by nicotine as indicated by a dramatic decrease of total protein level of YAP1, particularly the dephosphorylated YAP1 (Figure 1 F). This result suggests that the activation of YAP1 induced by nicotine is mediated through PKC.

### Knockdown of CHRNA3 also induced YAP1 activation

**Figure 2:**
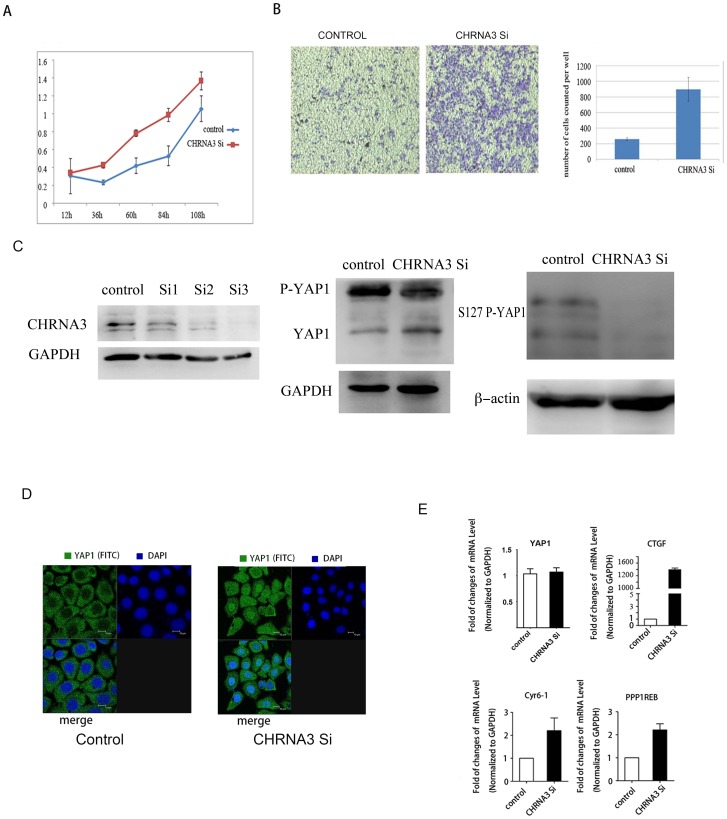
A. Cell growth curve measured with MTT assay indicated siRNA mediated knockdown of CHRNA3 increased the growth rate of KYSE510 cells. B. Transwell assay indicated that knockdown of CHRNA3 increased the invasion and migration of KYSE30 cells. C. Western blot analysis indicated 3 Stealth siRNA constructs effectively knocked down CHRNA3. Depletion of CHRNA3 led to a decrease of phosphorylated YAP1 and an increased of dephosphorylated YAP1, particularly a decreased S127 phosphorylation of YAP1. D. Observation of YAP1 subcellular localization with confocal fluorescence microscopy following depletion of CHRNA3. Translocation of YAP1 (green) from the cytoplasm to the nucleus was observed after siRNA mediated knockdown of CHRNA3 in KYSE510 cells for 48 h. E. Real-time PCR test indicated YAP1 targeted genes were induced by CHRNA3 knockdown in KYSE510 cell.

It has been shown that CHRNA5 (neuronal acetylcholine receptor subunit alpha-5) and CHRNA3 (neuronal acetylcholine receptor subunit alpha-3) as negative regulators of nicotine signaling in bronchial cancer and esophageal cancer cells [22]. Because knockdown of CHRNA3 and CHRNA5 increased the proliferation, migration and calcium influx of lung cancer cell lines, as a result of compensatory increase of assembly of α7-nAChR on the cytoplasm membrane which had higher permeability to calcium in response to nicotine. Thus we employed siRNA approaches to knockdown CHRNA3 in KSYE-510 cell and then examined the effects of CHRNA3 depletion on the growth and migration of KYSE510 cells, and on the activation of YAP1 as well. We observed an increase of growth rate and migration in KYSE510 cells by CHRNA3 knockdown, which is similar to that seen in the nicotine administration (Figure 2 A, Figure 2 B). Consistently, a decrease of YAP1 phosphorylation, particularly at the S127 site of YAP1 was shown by western blot (Figure 2 C). With confocal immunofluorescence microscope we also observed nuclear translocation of YAP1 in the KYSE510 cells following CHRNA3 knockdown (Figure 2 D). In addition, the transcriptional induction of CTGF and other YAP1 downstream genes were also observed in the cells silenced for CHRNA3 (Figure 2 E).

### Physical interactions between nAChRs and YAP1

**Figure 3:**
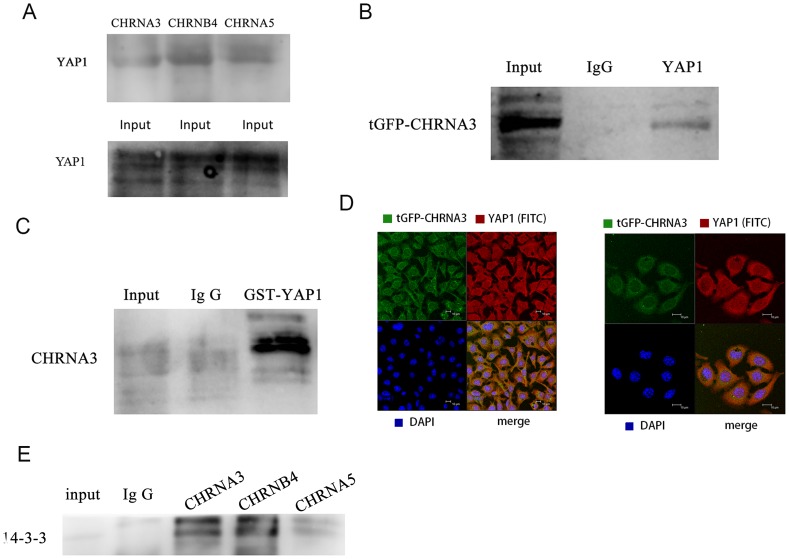
A. Endogenous IP test showed positive protein interactions between YAP1 and CHRNA3/CHRNB4/CHRNA5 in KYSE510 cells. B. Positive interaction between tGFP-CHRNA3 and YAP1 was detected in cells transfected with tGFP-CHRNA3. C. GST Pull assay indicated positive interaction of exogenously expressed GST-YAP1 with CHRNA3 in KYSE510 cell. D. Confocal immunofluorescence microscopy observed colocalization of YAP1 and CHRNA3 in KYSE510 cell. The KYSE510 cells were transiently expressed tGFP labeled CHRNA3 of TureClone vector (OriGene). YAP1 was labeled with red-FITC conjugated goat anti rabbit IgG. Cell nuclei were visualized with DAPI. E. endogenous IP detected positive interactions between 14-3-3 and CHRNA3/CHRNB4/CHRNA5.

To further explore the regulatory role of nAChRs with YAP1, we conducted immunoprecipitation experiments to investigate if nAChRs physically interact with YAP1. As shown in Figure 3 A, the clear interactions between YAP1 and CHRNA3/CHRNA5/CHRNB4 were identified. The interaction between YAP1 and CHRNA3 was verified with exogenously transfected tGFP-labeled CHRNA3 in KYSE510 cells (Figure 3 B). Additionally, we conducted GST pull down assay to further verify the interaction between YAP1 and CHRNA3 with exogenously expressed GST-labeled YAP1 (Abnova). GST-pull down experiment also showed with positive interaction between endogenous CHRNA3 and GST-YAP1 (Figure 3 C). Next, we used confocal immunofluorescence microscope to examine the colocalization between CHRNA3 and YAP1. We found that both CHRNA3 and YAP1 were colocalized at the membrane region and in the cytoplasm of KYSE510 cells (Figure 3 D). Collectively, these observations suggest nAChRs physically associate with YAP1 in esophageal cancer cells. 14-3-3 is the binding partner of YAP1 in the cytoplasm, we also observed positive interactions between 14-3-3 and CHRNA3/CHRNA5/CHRNB4 with immunoprecipitation assay in KYSE510 cell (Figure 3 E).

### Dissociation of the negative regulators of YAP1 by nicotine treatment

**Figure 4:**
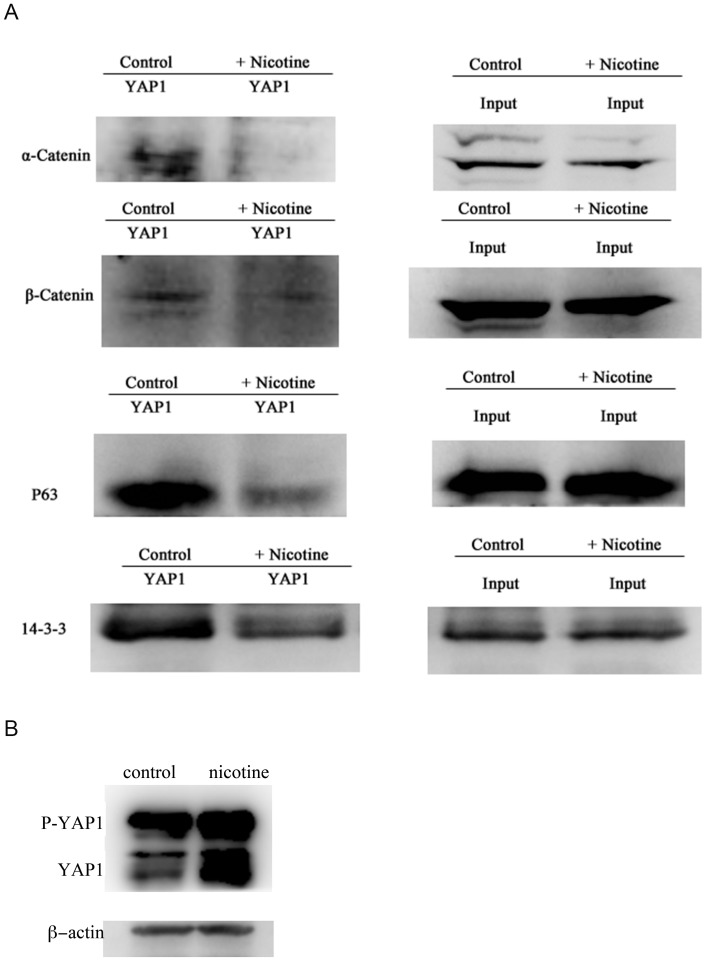
Protein Immunoprecipitation indicated a decreased physical association between YAP1 with 14-3-3, α-catenin, β-catenin and p63 upon nicotine administration at the concentration of 80 nM for 48 h in KYSE510 cells. B. KYSE510 cells exposed to nicotine for more than 4 days led to upregulation of total protein level of YAP1, including both the phosphorylated YAP1 and dephosphorylated YAP1 indicated by western blot analysis.

**Figure 5:**
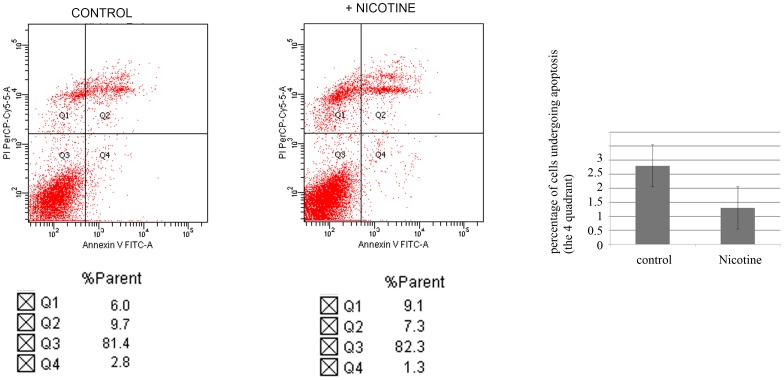
KYSE510 cells were treated with or without nicotine for 48(PI) were analyzed by flow cytometry. Nicotine treatment decreased the parent of apoptotic cells in the lower right quadrant.

The transcriptional activity and protein level of YAP1 is known to be negatively regulated by 14-3-3, α-catenin and β-catenin [8], [23], [24]. Phosphorylated YAP1 can bind with p63, and such association increases the stability of p63 and contributes to p63 mediated drug-induced apoptosis [21]. Thus we attempted to examine the effect of nicotine on the interactions of YAP1 with α-catenin, β-catenin, 14-3-3, and p63. As shown in Figure 4, the interactions of YAP1 with α-catenin, β-catenin, 14-3-3, and p63 were disrupted by nicotine administration (Figure 4 A), which is in accordance with our observations that nicotine treatment increased nuclear translocation and activation of YAP1. Decreased interaction of YAP1 with 14-3-3, α-catenin, β-catenin would result in an increase of total protein level of YAP1. Consistently, we have observed a significant increase of YAP1 protein level upon prolonged nicotine treatment (Figure 4 B). As p63 is an important regulator of apoptosis, and altered interaction between YAP1 and p63 would impair the apoptotic responses mediated by p63. Consistently, we observed decreased apoptosis of KYSE510 cells treated with nicotine (Figure 5).

### Immunohistochemistry analysis of YAP1 in clinical samples

**Figure 6:**
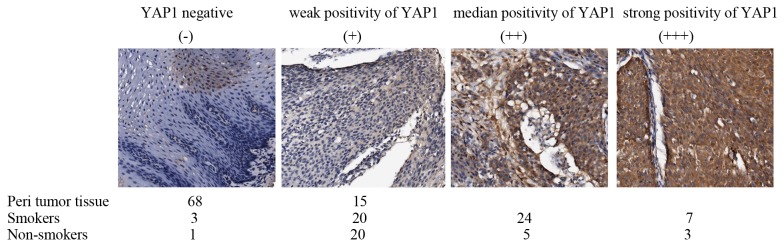
Immunohistochemistry analysis of esophageal cancer tissues with YAP1 antibody on microarray slides containing 83 patients of T3 esophageal squamous carcinoma. The slides were scanned by the AperioScanScope CS. Tissue samples were divided into 4 categories based on the intensity of YAP1 staining.

It has been shown that esophageal cancer patients with upregulation of YAP1 had a worse overall survival than those with normal expression of YAP1 [11]. Thus we used clinical esophageal cancer samples to examine the correlation between upregulated expression of YAP1 and cigarette smoking. We collected esophageal cancer samples from 83 patients at T3 stage, including 29 non-smokers and 54 smokers. We conducted immunohistochemistry experiment to analyze the expression level of YAP1 in the esophageal cancer samples, and found that the cancer patients with smoking history showed high expression of YAP1 compared with the non-smoking patients (*P*<0.05) (Figure 6 and Table 1). Thus, these finding go along with our observations that YAP1 was activated by nicotine administration in vitro. However, we did not observe a significant difference in the overall survivals between YAP1-high and YAP1-low groups, probably due to these cancer samples were from T3 stage esophageal cancers patients.

**Table 1:** YAP1 expression is positively correlated with smoking status of ESCC patients^1^.

	strong positivity of YAP1(+ + +)	median positivity of YAP1 (+ +)	weak positivity of YAP1 (+)	YAP1 negative (−)	Total cancer number
Non-Smokers^2^	3	5	20	1	29
Smokers^3^	7	24	20	3	54
Peri tumor tissue	0	0	15	68	83

(*P*<0.05, χ2 = 6.74).

1 83 ESCC clinical samples were examined for YAP1 expression using immunohistochemical approach as described in Materials and Methods.

2 Strong and median YAP1 staining in Non-smoker ESCC patients (8/29, 27.6%).

3 Strong and median YAP1 staining in smoker ESCC patients (31/54, 57.4%).

Correlations between the subgroups of staining and cigarette smoking were calculated using the Pearson and χ2 test.

## Discussion

Nicotine is an established oncogenic factor contributing to the pathogenesis of numerous cancers, including esophageal cancer [3], [4]. And nicotine is known to promote the proliferation of cancer cells through activation of the catecholamine signaling cascade [4], [25], [26]. Consistently we observed an increased growth rate for esophageal cancer cells by nicotine treatment or through knockdown of CHRNA3. Several groups have reported that nicotine exposure and cigarette smoking can promote the acquisition of cancer stem cell like and epithelial-mesenchymal transition in oral cancer, head and neck squamous cell carcinoma, lung cancer, and breast cancer [27]–[30]. Interestingly, Nallet-Staubet al. have demonstrated that YAP1 and TAZ contribute to the invasive and metastatic capacity of melanoma cells [31]. And Chen et al. reported YAP1 as an important mediator of TLR4/NANOG oncogenic pathway in maintaining the tumor-initiating stem-like cells (TICs) population by suppression of cytostatic TGF-β signaling in HCC [32]. These observations are in accordance with the early observation that YAP1 homolog TAZ is required for sustaining self-renewal and tumor-initiation capacities in breast cancer stem cells [33]. In this study, we have shown that nicotine administration or CHRNA3 depletion lead to an increase of cell growth and migration, and induce resistance to apoptosis in esophageal cancer cells.

G protein coupled receptors (GPCRs) can activate or inhibit the Hippo-YAP pathway depending on the coupled G proteins. And Lysophosphatidic acid (LPA) and sphingosine-1-phosphate (S1P) were reported to be the upstream agonists in this signaling pathway to activate YAP1/TAZ through regulating Lats kinase activity by modulating actin cytoskeleton dynamics [15]. GPCRs can activate calcium signaling cascade through PLC/IP3R/PKC pathway [34]. And calcium signaling is known to regulate actin dynamics and cell motility through the modulation of the downstream Rho GTPase signaling pathways [34], [35]. Thus nicotine may activate YAP1 through nAChRs mediated calcium influx. Interestingly, we observed PKC specific inhibitor Enzastaurin was able to block the activation of YAP1 by nicotine administration, which is consistent with PKC's role in potentiating calcium channels to enhancing calcium influx [36].

In this study, we determined the interactions between nAChRs and YAP1, indicating nAChRs mediated signaling may have a direct effect on YAP1 activation, which is further supported by observations that CHRNA3 co-localizes with YAP1. Furthermore, we observed altered physical associations between YAP1 and α-catenin/β-catenin/14-3-3 upon the activation of YAP1 by nicotine administration in esophageal cancer cell. It has previously been reported α-catenin and β-catenin physically interact with YAP1 to negatively regulated YAP1 activity and its degradation [23], [24]. The association between YAP1 and α-catenin is mediated by 14-3-3 and that IQGAP1 is one of the high confidence binding partners of YAP1 [23]. Interestingly, 14-3-3 interacts with nAChRs and tethers nAChRs into specific membrane domains through an interaction with a multi-subunit complex, comprising APC, EB1, IQGAP1 and MACF, which are anchored to the microtubule cytoskeleton [37]. 14-3-3 also promotes the forward transport of N-cadherin/β-catenin complexes from the ER [38]. In addition, β-catenin interacts with rapsyn to regulate the clustering of nAChRs in muscle cells [39]. In this study, we observed positive interactions between 14-3-3 and CHRNA3/CHRNA5/CHRNB4 in KYSE510 cell. These findings suggest nAChRs, 14-3-3, IQGAP1, α-catenin and β-catenin may form a cytoskeleton anchored negative regulation complex with YAP1, and 14-3-3 serves as the common binding adaptor of the complex. This complex negatively regulates YAP1 activation and nuclear translocation and tether YAP1 to the cytoskeleton in the cytoplasm. Thus, nAChRs regulate the activation of YAP1 through dissociation of the complex in response to upstream signals.

Interestingly we also observed a decreased P63 association with YAP1 by nicotine administration, which promotes p63 degradation and inhibit apoptosis. JNK phosphorylation of YAP1 is required to stabilize p63 through direct binding with YAP1 [21]. Nicotine signaling is known to inhibit JNK1 activity in cancer cells [40]. Whereas, disruption of actin cytoskeleton can activate JNK/SAPK pathway to stabilize p21 monitored by the hippo pathway upstream kinase MST1 and MST2 [41]. Likely, nicotine induced cytoskeletal remodeling mediated by nAChRs inhibits JNK activity, leading to decreased binding of YAP1 with p63. Moreover, nicotine also induces upregulation of XIAP and survivin to inhibit apoptosis, survivin is a conserved downstream gene of YAP1 [13].

Upregulation and increased nuclear translocation of YAP1 have been reported in esophageal cancers. Particularly upregulation of YAP1 in the nucleus is significantly associated with poor overall survival [11]. In the immunohistochemical analysis we examined 83 T3 stage esophageal cancer samples including 29 non-smokers and 54 smokers, among which we found 49 cancer samples with median or strong upregulation of YAP1 (60%). Importantly, we found a significant association between YAP1 upregulation and cigarette smoking, indicating a causal relationship between cigarette smoking and the oncogenic activation of YAP1 in esophageal cancer. Because YAP1 is also activated in many other types of cancers, the oncogenic activation of YAP1 induced by smoking may be a common mechanism for the carcinogenic effects of cigarette smoking. In summary, these findings suggest nAChRs function as upstream regulators of the hippo pathway, and nAChR-signaling can induce nuclear translocation and activation of YAP1 upon nicotine exposure. Thus we discovered a novel link between cigarette smoking and the oncogenic activity of YAP1 in esophageal cancer. Targeting on molecules involved in nAChR-signaling and Hippo pathway such as YAP1 and PKC may represent a promising strategy for the treatment of smoking related cancers.
